# Mapping the small-world properties of brain networks in deception with functional near-infrared spectroscopy

**DOI:** 10.1038/srep25297

**Published:** 2016-04-29

**Authors:** Jiang Zhang, Xiaohong Lin, Genyu Fu, Liyang Sai, Huafu Chen, Jianbo Yang, Mingwen Wang, Qi Liu, Gang Yang, Junran Zhang, Zhen Yuan

**Affiliations:** 1 Department of Medical Information Engineering, School of Electrical Engineering and Information, Sichuan University, Chengdu 610065, China; 2 Bioimaging Core, Faculty of Health Sciences, University of Macau, Macau SAR China; 3 Department of Psychology, Hangzhou Normal University, Hangzhou 311121, China; 4 Department of Psychology, Zhejiang Normal University, Jinhua 321004, China; 5 School of Psychology & Cognitive Science, East China Normal University, Shanghai 200062, China; 6 School of Life Science and Technology, University of Electronic Science and Technology of China, Chengdu 610054, China; 7 College of Applied Nuclear Technology and Automation Engineering, Chengdu University of Technology, Chengdu 610059, China; 8 School of Mathematics, Southwest Jiaotong University, Chengdu, 610031, China

## Abstract

Deception is not a rare occurrence among human behaviors; however, the present brain mapping techniques are insufficient to reveal the neural mechanism of deception under spontaneous or controlled conditions. Interestingly, functional near-infrared spectroscopy (fNIRS) has emerged as a highly promising neuroimaging technique that enables continuous and noninvasive monitoring of changes in blood oxygenation and blood volume in the human brain. In this study, fNIRS was used in combination with complex network theory to extract the attribute features of the functional brain networks underling deception in subjects exhibiting spontaneous or controlled behaviors. Our findings revealed that the small-world networks of the subjects engaged in spontaneous behaviors exhibited greater clustering coefficients, shorter average path lengths, greater average node degrees, and stronger randomness compared with those of subjects engaged in control behaviors. Consequently, we suggest that small-world network topology is capable of distinguishing well between spontaneous and controlled deceptions.

Deception is an interesting linguistic, psychological and social behavior of human beings. Identifying the features of deception using neuroimaging techniques is essential for revealing the complex cognitive mechanisms that underlie lying. Electroencephalography (EEG) and event-related potential (ERP) are the primary neurophysiologic tools that have been used in the evaluation of deception^1^,^2^. Interestingly, in addition to ERP/EEG, functional near-infrared spectroscopy (fNIRS) provides another option that involves optically^3^ monitoring and imaging cerebral blood oxygen and hemoglobin concentration changes related to lying^4^,^5^,^6^,^7^,^8^. Compared to the available functional neuroimaging modalities, including functional magnetic resonance (fMRI) and positron emission tomography (PET), fNIRS offers unsurpassed temporal resolution and provides quantitative hemodynamic information about both oxyhemoglobin (*HbO2*) and deoxyhemoglobin (*HbR*)^9^,^10^. Importantly, fNIRS can be implemented in the form of a wearable and noninvasive or minimally intrusive device that has the capability to monitor brain activity in real-life conditions and in everyday environments. Obtaining measurements of the hemodynamic responses of localized regions of the brain allows for inferences to be made regarding the neural correlates of deception.

Although neural mechanism-oriented investigations of deception relative to honesty have been conducted using fNIRS in recent years^8^,^11^,^12^,^13^,^14^,^15^, the majority of this work completed focused on detecting areas of brain activity^8^,^11^,^12^,^13^,^14^,^15^. These studies have unanimously identified significant brain activities occurring in the prefrontal cortex (PFC) and the anterior cingulate cortex (ACC) in cases of deception^8^,^11^,^12^,^13^,^14^. However, advances in the study of neural mechanisms are eliciting a transition from revealing the regions of brain activity to identifying the brain networks. Specifically, complex network theory is developing into a powerful analysis tool for constructing brain networks. Complex networks can explore the topological relations of nodes and edges owing to small-world and scale-free characteristics^19^,^20^. To date, complex network analysis has been performed in many fields^16^,^17^,^18^, and small-world or scale-free properties have been identified in many real networks^16^,^17^,^18^,^19^,^20^,^21^. More importantly, recent work on functional brain connectivity has revealed that the brain networks underlying cognitions and neurological disorders also have small-world statistical properties^16^,^21^,^22^,^23^,^24^,^25^,^26^,^27^.

In this study, we sought to investigate whether the functional brain networks of deception also have small-world properties. We studied the topological and spatial features of local brain networks in terms of the deceptions underling spontaneous and controlled behaviors. Specifically, we analyzed the functional brain networks of deception generated as revealed in fNIRS recordings using indices of small-world network characteristics, including the clustering coefficients, average path lengths and average node degrees of the brain networks. Because the small-world properties of neural networks regarding deception during spontaneous and control behaviors have not been explored, this pilot work will definitely pave a new avenue for an improved understanding the cognitive mechanism underlying lying.

## Materials and Methods

### Subjects

Twenty-four right-handed subjects (11 males, mean age: 19–22 years) participated in this study. Participants with reported histories of neurological or psychiatric disorders were excluded from the study. All subjects were required to sign informed consent documents prior to the experimental tests. All the clinical tests were approved by the Ethics Committees of Zhejiang Normal University and the University of Macau and were carried out in accordance with the approved guidelines.

### Tasks

This experiment included two tasks: a spontaneous deception task and a control deception task. During the experiment, the subjects were asked to play a multiple-round poker game via a computer with an opponent who was in a separate single room.The participant who acquired more points won the game.

The spontaneous deception task always began first, and during the stimuli period, the winner could randomly be rewarded with different amounts of money (0.5 Chinese yuan (CNY), 1 CNY or 2 CNY). Notably, in the spontaneous task, only the opponent was able to pick up the first card in each round of the poker game, and only the subject was able to see both his (her) and the opponent’s cards. Consequently, the subject knew exactly who acquired more points in each round and was required to send the final results of the competition (answers) to the opponent each round. When the subject won the game, he (she) most frequently sent the correct answer (win-win) to the opponent. However, when the subject lost the game, he (she) was able to send a false answer to the opponent (lose-win) to receive a reward by deception or send the correct answer and lose the poker game (lose-lose). During the entire spontaneous stimuli period, the subject was only able to win ten of the 40 rounds of the poker game; for the remaining 30 rounds, the subject was able to choose to lie or be honest. The test cases of spontaneous behavior were provided in Table 1.

The control deception tasks were similar to the spontaneous task except that the subjects were required to follow instructions on the screen of the computer regarding whether to tell the truth or lie each round. Moreover, in the control tasks, there were no rewards for the winner. During the stimuli period, the subjects were instructed to pass the right or wrong answers to the opponent (i.e., the truth or a lie). The control task consisted of three conditions: if the subjects won the poker game, they were required to send the right answer (win-win) to the computer; and if the subjects lost the game, they were required to send the false (lose-win) or the true answer (lose-lose) to the computer. The tasks included 10 rounds of each of the three conditions (i.e., win-win, lose-win, and lose-lose), and test cases of the control behavior were also provided in Table 1.

### Data Acquisition

**Figure 1:**
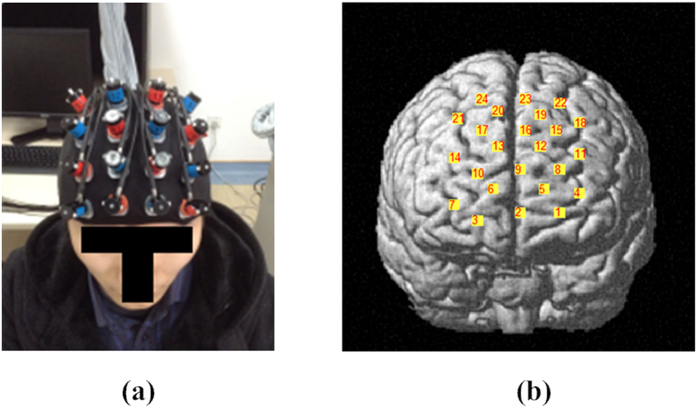
(**a**) The fNIRS system. (**b**) The locations of the 24 NIRS channels along the cortex. Panel (**b**) was generated by introducing the data collected with the 3D-magnetic space digitizer into the statistical parameter mapping tool box for NIRS (NIRS-SPM: http://bispl.weebly.com/nirs-spm.html#/)^33^.

A 24-channel continuous wave fNIRS system (ETG-4000, Hitachi Medical Co., Japan) with eight light emitters and eight detectors was utilized for the present experimental tests (see Fig. 1(a)). The optodes of the fNIRS systems were fixed using a single 9-cm × 9-cm rubber shell placed along the frontal areas. The shell was covered with a nylon net to ensure that the optodes effectively contacted the scalp. The 16-probe shell was arranged in a 4 × 4 array and was capable of measuring the changes in the concentrations of hemoglobin across the 24 channels. The placements of the probes in the dorsal bilateral frontal areas were based on the findings of previous work^8^,^12^,^28^,^29^. The inter-optode distance was 30 mm, which allowed for measurements of the neural activities occurring approximately 15–25 mm beneath the scalp. The optical datasets from each individual channel were collected at two different wavelengths (695 and 830 nm) and further analyzed using the modified Beer-Lambert law for highly scattering media^8^,^30^. Changes in the concentrations of oxygenated (HbO2) and deoxygenated hemoglobin (Hb) were recorded in the units of millimolar-millimeter (mM × mm)^31^, and the sampling rate was set to 10 Hz.

A 3D-magnetic space digitizer (EZT-DM401, Hitachi Medical Corporation, Japan) was used to capture the three-dimensional spatial information of each optode on each participant’s scalp. We used the probabilistic registration method from NIRS-SPM^32^,^33^ to estimate each channel’s corresponding location in the Montreal Neurological Institute (MNI) space (http://bispl.weebly.com/nirs-spm.html#/). The locations of the 24 channels along the cortex were illustrated in Fig. 1(b).

### Data Pre-processing

**Figure 2:**
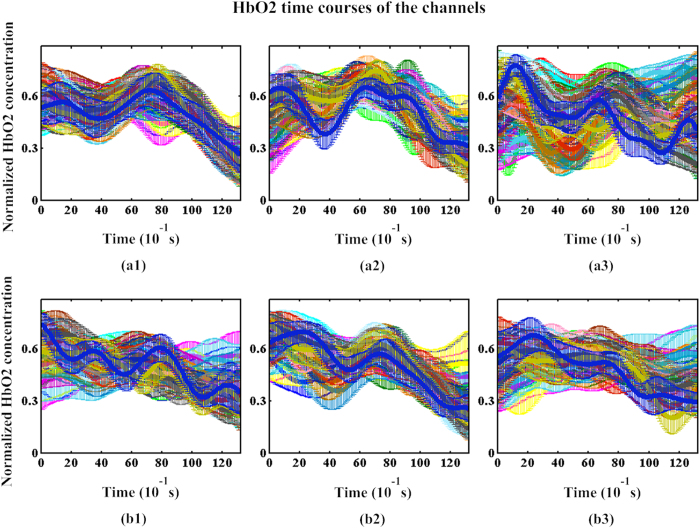
HbO2 measurements (mean ± variance) across all the subjects from the 24 channels. (**a1**–**a3**) The spontaneous behavior cases from all the channels: (**a1**) win-win, (**a2**) lose-win, and (**a3**) lose-lose. (**b1**–**b3**) The control behavior cases from all the channels: (**b1**) win-win, (**b2**) lose-win, and (**b3**) lose-lose. The different colors of the curves represent the HbO2 signals from the different channels, the vertical axis represents the normalized HbO2 concentration changes and variances, and the horizontal axis represents the time (unit: 10^−1^ second).

In this experiment, the Hb and HbO2 datasets were first processed using a 0.01-Hz high-pass filter and a 0.3-Hz low-pass filter^8^,^34^. Next, the datasets were segmented in relation to different markers that included three types of markers for the spontaneous behaviors and three additional types of markers for the control behaviors (i.e., the win-win, lose-win, and lose-lose behaviors). The only difference between the spontaneous deception and control deception tasks was that during the control behaviors, the participants were required to follow the computer’s instructions regarding telling the truth or lying rather than making the decision by themselves. The duration for each run of 13 s, which included a 2-s pre-stimulus period and an 11-s post-stimulus and recovery period. Because changes in HbO2 concentration are widely recognized as the most sensitive indicators of the brain’s hemodynamic responses^6^,^8^,^35^, the HbO2 data from all the channels of all subjects were analyzed. The mean results and variances of the HbO2 signals from all subjects were presented in Fig. 2. The run averages of the normalized HbO2 data were first calculated for each channel, and then the grand-average results and variances for all 24 subjects were calculated for both the spontaneous and control behaviors and were illustrated in Fig. 2.

### Correlation Coefficients and Binary matrixes

To generate a brain network, it is essential to define the nodes and edges of the network. In the present study, a node was defined as an fNIRS channel, and an edge was specified as the Pearson correlation coefficient of the measurements from any two channels. Importantly, the correlation coefficient of two time series was considered to be indicative of the functional connectivity between two nodes of the brain network^36^,^37^. To analyze the properties of the network topology constructed from all nodes based on graph theory, we computed the correlation coefficients between all 24 channels and then generated the correlation coefficient matrixes. During the process of constructing the brain networks, we had to binarize the correlation coefficient matrix by setting a threshold *T*. Consequently, the element value of the matrix was set to 0 when the absolute value of the Pearson correlation coefficient was smaller than *T* and was not considered indicative of significant connectivity, and the corresponding edges were thus viewed as non-existent in the network analysis. In contrast, the element value was set to 1 when the absolute value of the correlation coefficient was larger than or equal to the *T*^38^. In this manner, binary matrixes between the pairs of nodes were acquired. To further facilitate the conversion of the binary matrix into a visualizable spatial network, the values of the binary matrix can be mapped into three-dimensional (3D) space as the edges of the brain network using the BrainNet Viewer tool (http://www.nitrc.org/projects/bnv/)^39^.

### Characteristic Indices of the Network

For complex networks, the clustering coefficient of the network, the average path length, the average node degree and the small-world network measure are often used in network topology characteristic analyses. After acquiring the binary matrix of nodes, these indicators can be directly obtained.

The clustering coefficient of the network is defined as follows^17^,^40^:


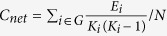
, where *N* is the number of nodes, *G* is the set of all nodes in the network, 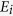 is the number of edges in the subgraph 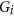, and 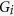 is defined as the graph including the nodes that are the direct neighbors of the ith node, i.e., those nodes that are directly connected to the ith node with an edge^19^. 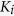 is the number of nodes that are directly connected to the node *i*, which is defined as the degree of node *i*. Moreover, the average node degree of the network^40^, i.e., 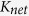, is defined as the mean of the degrees of all the nodes within the network.

The average path length of network is defined as follows^17^,^40^:


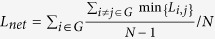
, where min 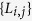 is the shortest path (geodesic) between node *i* and node *j*. The small-world network measure is defined as 
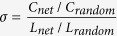
40,41, where 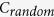 and 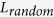 are the clustering coefficient and average path of the random network and correspond to the clustering coefficient 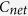 and average path length 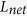 of the brain network, respectively. Further, we adopted the Markov chain algorithm to generate 100 random networks with degree sequences that correlated with the binary matrix. Next, we considered the mean clustering coefficients and average path lengths of the 100 random networks as the clustering coefficient and average path of the random network, respectively^19^,^38^,^42^,^43^,^44^.

## Results and Discussion

In this fNIRS experiment, a digitizer was used to quantify the 3D spatial locations of the probes and the channels, and each channel’s corresponding location in the MNI space was estimated and identified according to the international 10–20 system for EEG. Based on these results, we were able to ensure that the probes were placed correctly in the different regions for the capture of all the brain activity. Moreover, the behaviors of the subjects in both the spontaneous or control conditions corresponded to the three cases as illustrated in Table 1. Next, we investigated the relationships and attributes of the constructed networks in relation to the local brain activity changes during the three cases of both the spontaneous and control behaviors.

**Figure 3:**
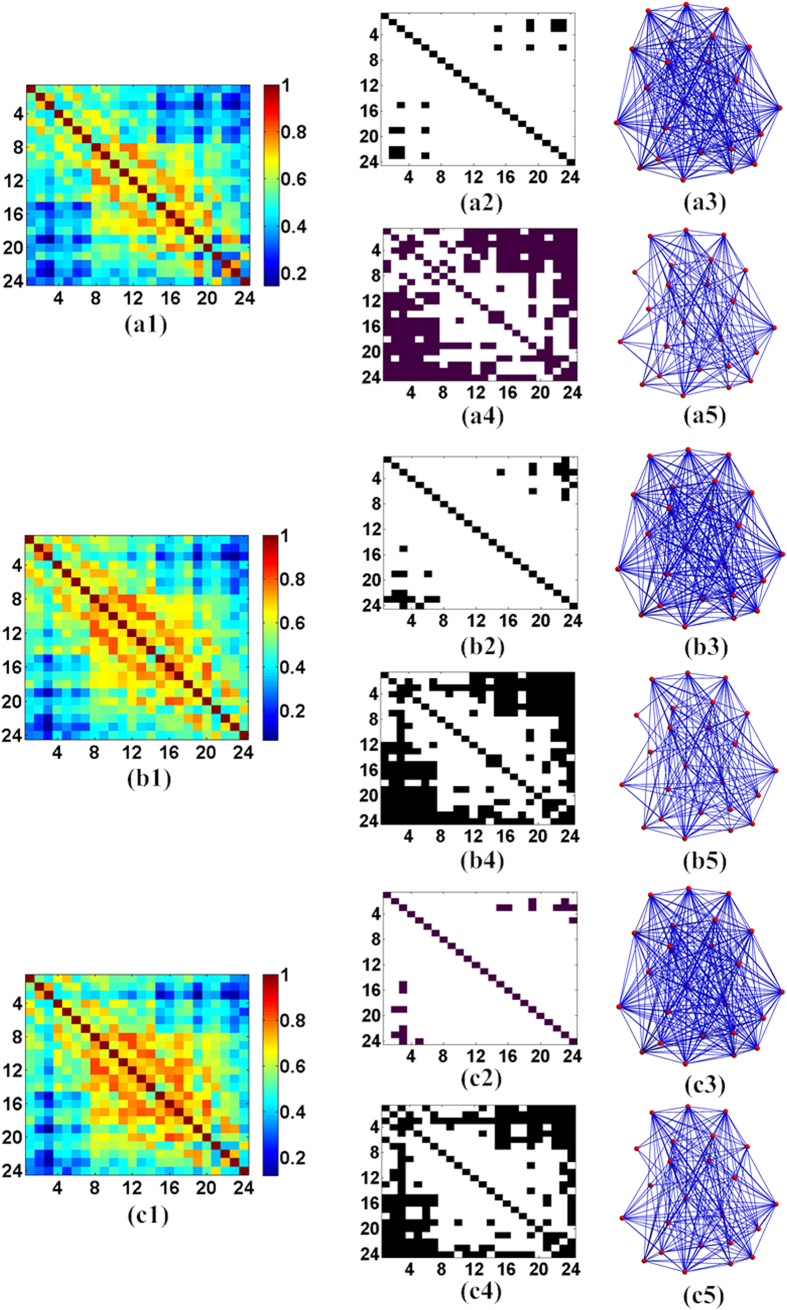
Correlation coefficients, binary matrixes and 3D relative networks of the brain nodes for the spontaneous behavior in the three cases: (**a1–a5**), case 1; (**a2,a3**), the results with the threshold value of 0.275; and (**a4,a5**), the results with the threshold value of 0.5. (**b1–b5**) Case 2: (**b2,b3**), the results with the threshold value of 0.275; and (**b4,b5**), the results with the threshold value of 0.5. (**c1–c5**) Case 3: (**c2,c3**), the results with the threshold value of 0.275; and (**c4,c5**), the results with the threshold value of 0.5.

Fig. 3(a1)–(a5) presented the computed Pearson correlation coefficient matrix, binary matrix and 3D relative networks of the brain nodes, respectively, for case 1 in Table 1 in addition to the spontaneous behavior. Specifically, Fig. 3(a1) displayed the averaged correlation coefficients from all the subjects. The horizontal and vertical axes in this figure denote the nodes, and the elements within the square areas represent the correlation coefficients between the nodes. Additionally, the binary matrixes in Fig. 3(a2),(a4) were generated with threshold values of 0.275 and 0.5 as applied to the results displayed in Fig. 3(a1) in which the white areas represent significant connections. Further, Fig. 3(a3),(a5) provided a 3D visualization of the networks for the binary matrixes presented in Fig. 3(a2),(a4), respectively. For the spontaneous behavior of case 2, Fig. 3(b1)–(b5) contained plots of the Pearson correlation coefficient matrix, binary matrix and 3D relative networks of the brain nodes, respectively. Moreover, the elements of the coefficient matrix in Fig. 3(b1) provided the mean Pearson correlation coefficients between the nodes of all 24 subjects. The binary matrixes in Fig. 3(b2),(b4) presented the results of the binarization of the elements in Fig. 3(b1) at the threshold values of 0.275 and 0.5, respectively. Similarly, Fig. 3(c1)–(c5) presented the Pearson correlation coefficient matrix, binary matrix and 3D relative networks of the brain nodes, respectively, for case 3 during the spontaneous behavior.

**Figure 4:**
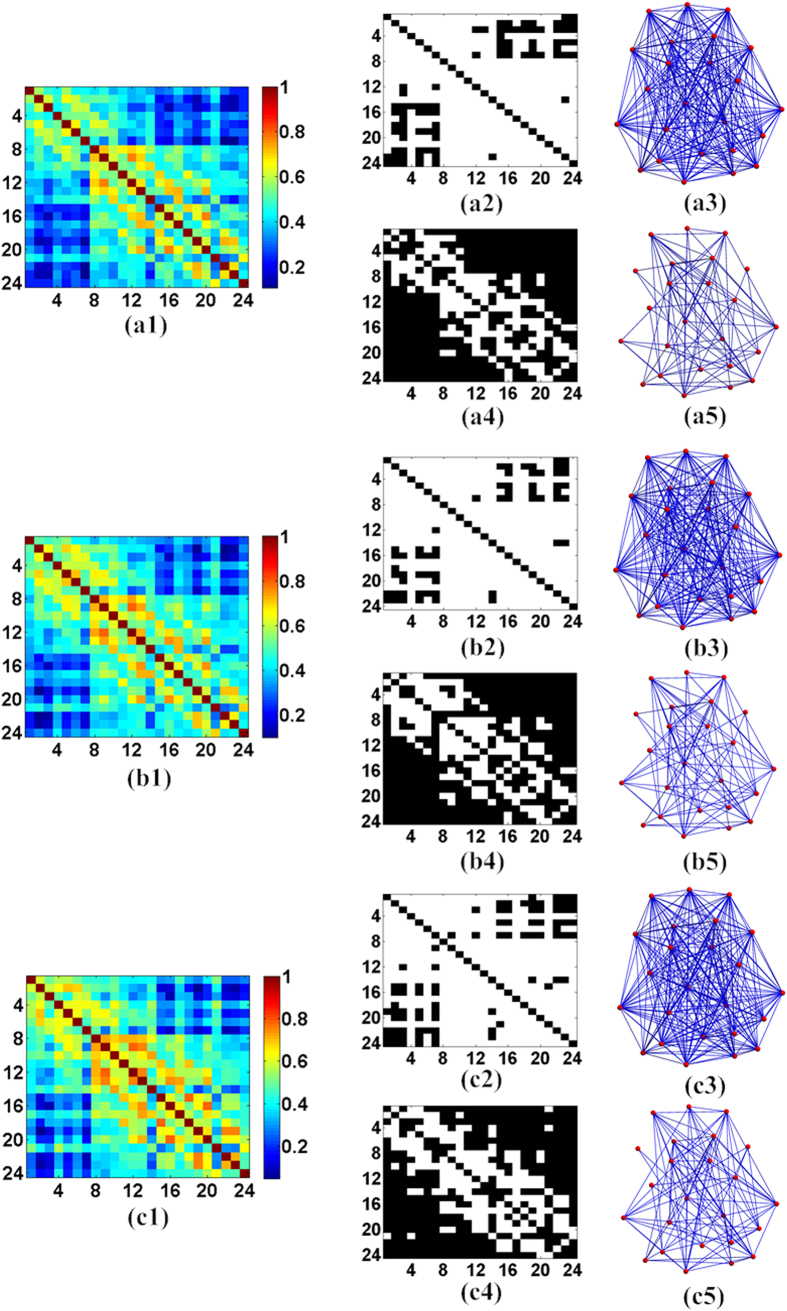
Correlation coefficients, binary matrixes and 3D relative networks of the brain nodes for the control behavior in the three cases: (**a1–a5**), case 4; (**a2,a3**), results with the threshold value of 0.275; and (**a4,a5**), results with the threshold value of 0.5. (**b1–b5**) Case 5: (**b2,b3**), the results with the threshold value of 0.275; and (**b4,b5**), the results with the threshold value of 0.5. (**c1–c5**) Case 6: (**c2,c3**), the results with the threshold value of 0.275; and (**c4,c5**), the results with the threshold value of 0.5.

For the control behavior of case 4, Fig. 4(a1)–(a5) presented the Pearson correlation coefficient matrix, binary matrix and 3D relative networks of the brain nodes, respectively. Again, the elements of the coefficient matrix in Fig. 4(a1) were the mean Pearson correlation coefficients from the 24 subjects. The binary matrixes in Fig. 4(a2),(a4) were generated with the threshold values of 0.275 and 0.5, respectively. Finally, Fig. 4(b1)–(b5),(c1)–(c5) presented the results of the analyses of cases 5 and 6 during the behaviors, respectively. Interestingly, it can be observed from Figs 3 and 4 that the brain networks for the spontaneous behaviors exhibited more edges than those for the controlled behaviors. We also found that the connections between the pairs of nodes seemed to be more active during the spontaneous behavior than during the control behavior.

**Figure 5:**
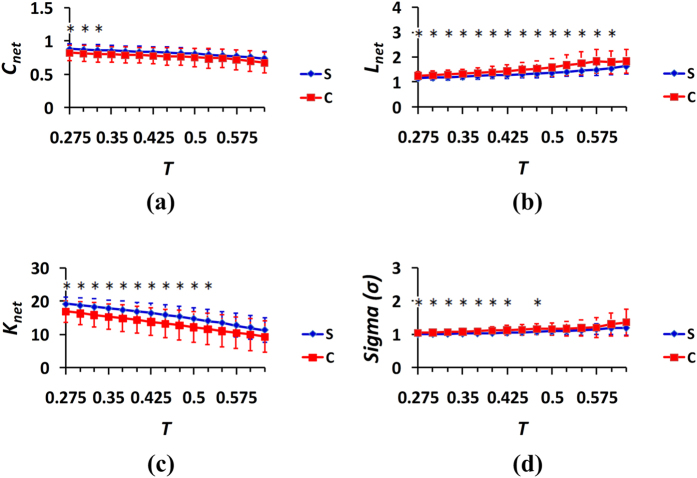
Properties of the brain networks under the spontaneous and control behavior conditions (cases 1 and 4 in Table 1): (**a**) clustering coefficient; (**b**) average path length; (**c**) average node degree; and (**d**) measure of small-worldness. The curves (mean ± SD) indicate the network indicators at the different thresholds. The blue color represents the spontaneous behavior, and the red color represents the control behavior. The horizontal axes denote the threshold values, and the vertical axes denote the network property indexes. *p < 0.05 (the p values are from two-sample t-tests between the spontaneous and control behaviors at various threshold values).

**Figure 6:**
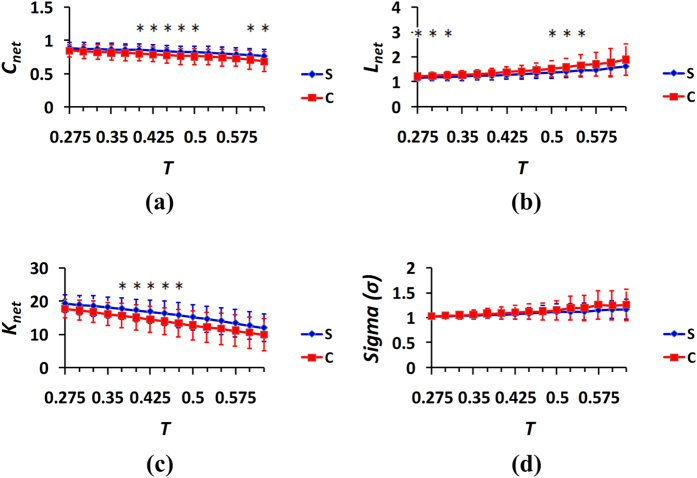
Properties of the brain networks in the spontaneous and control behavior conditions (cases 2 and 5 in Table 1): (**a**) clustering coefficient; (**b**) average path length; (**c**) average node degree; and (**d**) measure of small-worldness. The curves (mean ± SD) indicate the network indicators at the different thresholds. The blue color represents the spontaneous behavior, and the red color represents the control behavior. The horizontal axes denote the threshold values, and the vertical axes denote the network property indexes. *p < 0.05.

**Figure 7:**
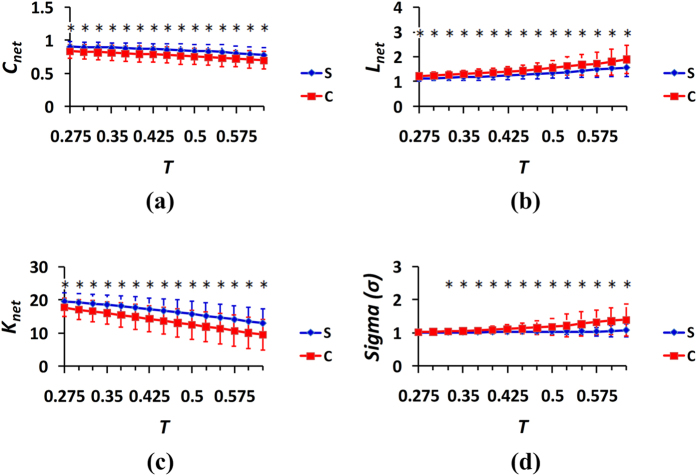
Properties of the brain networks in the spontaneous and control behavior conditions (cases 3 and 6 in Table 1): (**a**) clustering coefficient; (**b**) average path length; (**c**) average node degree; and (**d**) measure of small-worldness. The curves (mean ± SD) indicate the network indicators at the different thresholds. The blue color represents the spontaneous behavior, and the red color represents the controlled behavior. The horizontal axes denote the threshold values, and the vertical axes denote the network properties indexes. *p < 0.05.

The characteristics of the network are affected by the threshold values; however, there is not yet a unified standard for selecting the threshold value^19^. In the present study, we set the minimum threshold value 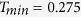 to ensure that the Pearson’s correlation coefficients were significant^19^.Moreover, we set the maximum threshold value 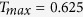 based on the condition that the networks meet the connectivity of the nodes (i.e., 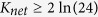)^20^,^38^. Therefore, the scope of threshold *T* for the correlation coefficients [0.275, 0.625] and a step size of the threshold of 0.025 were used to detect the network topological properties^19^,^20^,^38^,^45^. For the three cases of the spontaneous and control behaviors, the captured properties of the generated networks were compared, and the results were provided in Figs 5, 6, 7 in which the curves illustrated the distributions of the mean values and standard deviations (SDs) from the 24 subjects.The blue color represents the spontaneous behaviors, and the red color represents the control behaviors.The results of the two-sample t-tests between the spontaneous and control behavior groups were analyzed at different thresholds, and the significant differences were provided in Figs 5, 6, 7.

For the win-win condition (case 1 of the spontaneous behavior and case 4 of the control behavior), from Fig. 5(a), it can be seen that the network clustering coefficients of the spontaneous behavior were higher than those of the control behavior at all the threshold values. However, the average path lengths of the networks from the control behavior were larger than those of the spontaneous behavior for all threshold values as plotted in Fig. 5(b). Additionally, the average node degrees of the network for the spontaneous behavior illustrated in Fig. 5(c) were greater than those of the network for the control behavior. The greater clustering coefficient and greater average node degree of the network for the spontaneous behavior revealed that the brain nodes of the spontaneous behavior network exhibited much closer and denser connections than the network of the control behavior, whereas the lower average path length indicated that the spontaneous behavior included more efficient connections. If a network has small-world properties, the measure of small-worldness should be larger than 1^46^,^47^. Fig. 5(d) demonstrated that the local brain networks for both the spontaneous and control behaviors had small-world characteristics; however, the small world property index of the control behavior was greater than that of the spontaneous behavior. The small-world properties increased with increases in the threshold values.

The clustering coefficient, average path lengths, average node degrees, and small-worldness measures of the networks were computed and compared at different thresholds between cases 2 and 5. The findings were displayed in Fig. 6. Similarly, judging from the three network metrics, i.e., the clustering coefficient, average path length and average node degree of the network, the neural findings from the spontaneous behavior indicated that the local brain networks exhibited greater efficiencies and denser connections than those of the networks for the control behavior. The means of the measures of small-worldness for the spontaneous behavior ranged from 1.022 to 1.171, whereas the corresponding values of the control behavior ranged from 1.030 to 1.261, which indicated that the local brain activity networks of deception had small-world characteristics in both the spontaneous and control behavior states, and the small world property index of the control behavior was greater than that of the spontaneous behavior.

Again, the clustering coefficients, average path lengths, average node degrees, and measures of small-worldness of the network were compared at different thresholds between cases 3 and 6, and the results were provided in Fig. 7. Interestingly, it can be observed from Fig. 7 that based on the findings regarding the three network metrics, i.e., the clustering coefficient, average path length and average node degree of the network, the spontaneous behavior exhibited greater strength in terms of the local density or cliquishness of the information transfer in the network, greater efficiency and greater brain connectivity density between the nodes of the networks. The local brain networks were also found to have the small-world characteristics in both the spontaneous and control behavior states, and the small world property index of the control behavior was greater than that of the spontaneous behavior.

In complex network systems, the clustering coefficient of the network is indicative of the local efficiency or small groupness of information transfer in the network^46^. The average path length of the network describes the global efficiency and the ability for parallel information transmission in the network, and large average path lengths correlate with low efficiencies^46^. The average node degree represents the network density; when the network connections are sparse, the average node degree is small, and when the network connections are dense, the average node degree is large^46^. The measure of small-world signals indicates the small-world properties of brain functional networks^19^,^48^. As can be observed in Figs 5, 6, 7, the brain networks of the spontaneous behavior exhibited greater clustering coefficients, shorter average path lengths, greater average node degrees and weaker small-world properties (i.e., greater randomness) compared with the networks of the control behavior. Consequently, we concluded that the functional networks of brain activity for the spontaneous behavior exhibited greater aggregation, efficiency and randomness during deception than during the control behavior.

The comparisons of the measures of small-worldness in Figs 5(d) and 7(d) indicate that the differences in the small-world properties of the brain activation networks between the control and spontaneous behaviors were the most significant when the real answer was “lose,” and the subjects’ answer was also “lose.” Additionally, there were also more significant differences in the property indexes of the brain networks between the conditions of the spontaneous and control behaviors illustrated in Fig. 7 compared with those illustrated in Figs 5 and 6. These findings indicate that when the real answer was “lose,” the brain appeared to exhibit more subconscious activities of some type in the “control” and forced “lose” choice cases than in the cases of spontaneous choice.

Notably, although the fNIRS system has demonstrated some unbeatable advantages in clinical neuroimaging, it also has limitations. For example, because of the strong optical absorption of hemoglobin, the penetration depth of fNIRS is generally less than 3 cm (between 2 and 3 cm). Specifically, owing to the strong photon scattering, the imaging resolution of fNIRS is around 6 mm, which is much lower than that of fMRI. In contrast, compared with fMRI and EEG techniques, fNIRS has the advantages of a relative insensitivity to movement artifacts because both the laser source and detectors are placed on the scalp and the relative distances between them are kept constant. Moreover, the effects of head movement can easily be eliminated using principle component analysis (PCA) or independent component analysis (ICA). However, in EEG and fMRI, PCA and ICA alone are insufficient to wholly remove the effect of movement artifacts without additional techniques.

## Conclusion

fNIRS is a tool that facilitates the exploration and building of functional brain networks. In this study, we combined this technology with complex network theory to extract and analyze the attribute features of functional brain networks in localized regions during deception under spontaneous and control behavior states. We discovered that the functional brain activation networks of the subjects (lying or telling the truth) underlying spontaneous behavior exhibited greater clustering coefficients, shorter average path lengths, greater average node degrees and stronger randomness than the networks underlying the control behavior. These findings revealed significant differences between the neural mechanisms of spontaneous and control deceptions from the perspective of the network.

## Additional Information

**How to cite this article**: Zhang, J. *et al.* Mapping the small-world properties of brain networks in deception with functional near-infrared spectroscopy. *Sci. Rep.* **6**, 25297; doi: 10.1038/srep25297 (2016).

## Figures and Tables

**Table 1:** Test cases of the spontaneous and controlled behaviors.

Type	Case	Real answer	Subjects’ answer	Result
Spontaneous behavior	1	win	win	truth
	2	lose	win	deception
	3	lose	lose	truth
Controlled behavior	4	win	win	truth
	5	lose	win	deception
	6	lose	lose	truth
