# Corrigendum: Mapping the small-world properties of brain networks in deception with functional near-infrared spectroscopy

**DOI:** 10.1038/srep26730

**Published:** 2016-05-31

**Authors:** Jiang Zhang, Xiaohong Lin, Genyue Fu, Liyang Sai, Huafu Chen, Jianbo Yang, Mingwen Wang, Qi Liu, Gang Yang, Junran Zhang, Zhen Yuan

Scientific Reports 6: Article number: 2529710.1038/srep25297; published online: 04292016; updated: 05312016

The original version of this Article contained a typographical error in the spelling of the author Genyue Fu, which was incorrectly given as Genyu Fu. This has now been corrected in the PDF and HTML versions of the Article.

